# Effect of Thickness on Color Stability of Gingiva-Colored Composite Resins Applied to 3D-Printed Resin

**DOI:** 10.3390/ma18204757

**Published:** 2025-10-17

**Authors:** Liliane da Rocha Bonatto Drummond, Isabela Reginaldo, Laís Duarte, Zuila Maria Lobato Wanghon, Analucia Gebler Philippi, Luiz Otávio de Oliveira Pala, Patrícia Pauletto, Thais Marques Simek Vega Gonçalves

**Affiliations:** 1 Department of Dentistry, Federal University of Santa Catarina (UFSC), Florianópolis 88040-900, Brazil; lilianebonatto@hotmail.com (L.d.R.B.D.); isareginaldo@gmail.com (I.R.); lais.drte@gmail.com (L.D.); wanghonzuila@gmail.com (Z.M.L.W.); analucia.p@ufsc.br (A.G.P.); thaisgonc@gmail.com (T.M.S.V.G.); 2 Department of Statistics, Institute of Exact Sciences and Technology, Federal University of Lavras (UFLA), Lavras 37203-202, Brazil; luizpala@ufla.br; 3 Department of Dentistry, Faculty of Dentistry, Universidad de las Americas (UDLA), Quito 170513, Ecuador

**Keywords:** gingiva-colored composites, color stability, 3D-printing, esthetics, in vitro studies, light-curing

## Abstract

Light-curing gingiva-colored composite resins (GCCs) are widely used for their esthetics and versatility, although they remain susceptible to discoloration. This in vitro study evaluated the effect of GCC thickness on color stability under different staining solutions and immersion times. Four hundred specimens were fabricated with a 3D-printed resin (P Pro; Institut Straumann AG), incorporating circular intaglio areas of varying thicknesses (0.2, 0.4, 0.6, 0.8, and 1.0 mm), into which paste (Nexco; Ivoclar AG) or flowable (Gradia Gum; GC Corp) GCCs were applied. After artificial aging in water at 55 °C for 5 days, specimens were immersed in coffee, black tea, red wine, or distilled water (control). Color differences (ΔE_00_) were assessed using digital photocolorimetry (eLAB protocol) and the CIEDE2000 formula at 2.5, 5, and 7 days. Data for each consistency were analyzed with 3-way repeated measures ANOVA and Tukey HSD (α = 0.05). Thicker GCCs (0.6–1.0 mm) showed significantly greater discoloration (*p* < 0.05). Flowable GCCs were more prone to color changes induced by coffee (*p* < 0.05), whereas paste GCCs exhibited more discoloration with black tea (*p* < 0.05). Extended immersion time increased color change, particularly in flowable GCCs. Overall, GCC thickness, immersion duration, and material consistency influenced long-term color stability.

## 1. Introduction

Polymethyl methacrylate (PMMA) has been widely used as a base material for removable dentures because of its good mechanical properties, biocompatibility, lightweight, versatility, and esthetics at relatively low cost [1]. However, this material has weaknesses such as brittleness, susceptibility to staining and wear, polymerization shrinkage, and the potential for allergic reactions and requires regular maintenance for long-term durability [2]. PMMA’s tendency to absorb water facilitates the diffusion of staining substances [1]. This can lead to noticeable color changes depending on factors such as prosthesis conditions, patient diet, and exposure to staining agents like beverages, smoking, or colored foods [3,4]. As a result, the esthetics of the prosthesis may be compromised [1,3].

With the advancement of digitally assisted dentistry, new techniques and materials have been developed to improve dental prosthesis manufacturing [5,6]. Milling techniques allow for more predictable processing, offering low technical sensitivity, high stability, excellent mechanical properties, and relatively good precision [5]. However, the milling process results in material waste and requires high-cost equipment to produce the prosthesis, especially a complete denture [5,6]. Additive manufacturing, however, minimizes waste, while allowing the creation of complex geometries at relatively low cost [6,7]. Therefore, studies [5,6,7] have been conducted to improve the mechanical properties and esthetics of 3-dimensionally (3D)-printed resins, aiming to enhance their overall performance [6].

Light-curing gingiva-colored composite resins (GCCs) offering excellent esthetics, durability, and customization have recently been introduced, enhancing prosthetic appearance and allowing repair by the dentist [8]. The esthetic results are predictable, as these products offer different shades and consistencies with immediate outcomes [9]. However, these products are susceptible to staining and tend to absorb moisture, which can degrade the material and increase color changes over time [10,11,12]. Furthermore, final esthetics depends on precise application in order to mimic the characteristics of the gingiva [10,11]. In addition, these materials present a certain degree of polymerization shrinkage and are generally more expensive than PMMA [10,11]. Moreover, most of the previous studies [13,14] had focused on tooth color composites, and more studies are needed to better understand the optical properties and long-term color behavior of the GCCs.

During prosthesis manufacturing, GCC has been commonly applied in different thicknesses to mimic the gingival characteristics; this may ultimately contribute to long-term color changes. According to Shamszadeh et al. [13] chromatic changes are related to the polymerization depth of the resins. When applied in thicker increments, the polymerization process may be compromised, leading to increased porosity and a greater susceptibility to surface color changes [13]. Conversely, in regions near the cervical margin, the reduced thickness of the composite layer may render it more susceptible to discoloration [11]. A previous study [14] reported that specimens of 3 mm showed greater chromatic changes when compared with those of 1 mm. However, these results were observed in tooth-colored resin materials, and data regarding the chromatic performance of GCC are still lacking.

Moreover, most of these studies evaluated color changes using visual methods, which are highly subjective [15,16]. In some studies [16,17,18], spectrophotometers and colorimeters were used. However, they require trained users for accurate results, and some equipment has been reported to be affected by environmental factors such as light, temperature, and humidity, making tooth color measurement in clinical settings challenging [15,17,19]. In contrast, digital photocolorimetry is a promising method that overcomes these limitations [20,21]. One example is the eLAB protocol, which is based on polarized photography that minimizes the influence of brightness and environmental conditions, providing precise color measurements while storing images, allowing for permanent records and comparisons over time [20,21].

Therefore, the aim of the present study was to evaluate the influence of GCC thickness on color differences (ΔE_00_) by varying the staining solution and immersion time, using a digital photocolorimetric method for color analysis. The null hypothesis was that no significant color differences (ΔE_00_) would be found between the different GCC thicknesses.

## 2. Materials and Methods

### 2.1. Specimen Preparation

**Figure 1:**
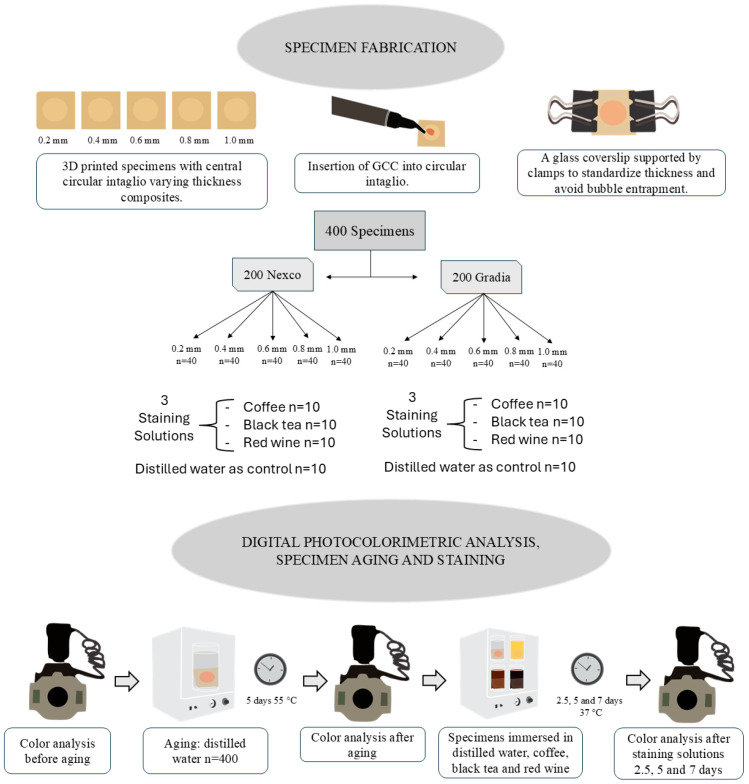
Flowchart of study methodology.

A total of 400 specimens were designed in a digital design software program (exocad Dental DB, v. 3.1; exocad GmbH) (18 × 18 × 2 mm). A central circular Ø = 10 mm intaglio was also designed, with varying thicknesses of 0.2, 0.4, 0.6, 0.8, and 1.0 mm, as shown in Figure 1. Then, all specimens were printed in a 3D printer (P20+; Institut Straumann AG, Basel, Switzerland) using a resin (P Pro; Institut Straumann AG) according to the manufacturer’s instructions for the preprocessing, processing, and postprocessing steps. Table 1 shows the components of each material used.

The sample size had been estimated from a pilot study, which was also performed to define key methodological aspects. Five specimens of each GCC thickness were printed and immersed in coffee staining solution. Color variation (ΔE_00_) was measured after 2.5, 5, and 7 days of immersion [22]. Based on the ΔE_00_ results of this pilot study, the sample size estimation was performed. Considering an effect size of 0.84, a minimum color variation difference of ΔE_00_ = 0.55 (±0.80), 80% power, and α = 0.05, 10 specimens per group were required to detect significant differences. In addition, the pilot study was used to calibrate two trained operators, ensuring methodological consistency before preparing the specimens from the real experiment.

After printing, specimens were randomly assigned to receive 1 of 2 GCC consistencies—flowable (Gradia GUM; GC Europe) or paste (Nexco; Ivoclar AG). The same two calibrated operators applied each GCC directly on the intaglio thickness immediately after surface treatment, as per the manufacturers’ instructions. Subsequently, a glass coverslip was placed over the applied GCC to control the layer thickness and prevent excess. This coverslip was secured laterally with 2 support clamps to avoid movement and air bubble entrapment (Figure 1). The specimens were light-cured for 30 s with an output of 1400 mW/cm^2^ and wavelength ranging of 440–480 nm using a light-curing unit (Valo Cordless Grand 3200; Ultradent Products, Inc., South Jordan, UT, USA), which was calibrated regularly with a dental light radiometer (Radiometer X, SDI, Victoria, Australia).

### 2.2. Staining Solutions

The color was analyzed before and after immersion in different staining solutions: coffee, black tea, and red wine (Figure 1). Distilled water was also used as a negative control for the staining solutions. The coffee solution was prepared using 60 g of coffee (3 Corações Tradicional; Tres, Santa Luzia, Brazil), filtered through a paper filter (Café Melitta; Melitta, Minden, Germany) with 600 mL of boiling mineral water. The black tea solution was prepared by immersing 7 tea bags (Chá Leão; Leão, Curitiba, Brazil) in 1 L of boiled water for 4 min. The red wine (Zanlorenzi, Campo Largo, Brazil) was used directly from the bottle at room temperature.

The specimens were placed in containers with their respective solutions and stored in an incubator at 37 ± 1 °C) for a 7-day period, with each solution changed daily. At the end of each immersion period (2.5, 5, and 7 days), the specimens were removed, rinsed under running water for 20 s, and dried with cold air jets for approximately 1 min [22]. Immediately afterward, the color was evaluated using the digital photocolorimetry method (eLAB protocol) [20] (Figure 1).

### 2.3. Digital Photocolorimetric Analysis

Standardized images of each specimen were obtained using a digital camera (Nikon D7200; Nikon Corp, Tokyo, Japan) according to the eLAB protocol [20]. The shutter speed was set to 1/125 s, with aperture value set to F = 22, and ISO sensor sensitivity at 200, with the image format set to RAW [20]. The focus adjustment was between 0.4 and 0.35, with an external circular flash attached and set to intensity level 1, along with a crossed polarizing filter (Polar_eyes; Emulation, Freiburg im Breisgau, Germany) [20]. A white balance card (White_balance; Emulation) and a black background were also used [20].

**Figure 2:**
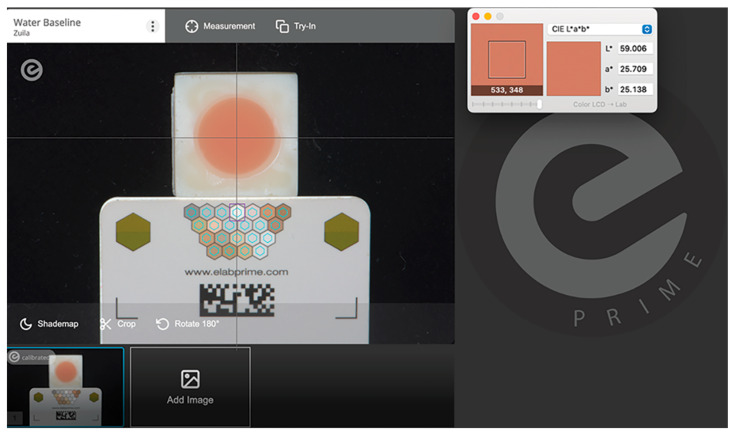
Standardized photography processed in the eLAB Prime software program (eLAB Prime; eLAB, version 3.0.18) and photographic coordinates measured in Classic Color Meter application.

Image processing was performed using the digital photographic processing software program (eLAB Prime; eLAB, version 3.0.18) (Figure 2). All evaluations were conducted by a single examiner who was not involved in specimen preparation or photography to minimize potential bias. The color recording was measured with the aid of the Commission Internationale de l’Éclairage (CIE) protocol using the L*a*b* (CIELab) color parameters [23,24]. The software application (Classic Color Meter application; version 2.1.1., Ricci Adams, Enola, PA, USA) was used to measure the colorimetric coordinates (L, a, b), which were recorded in a software program (Excel; Microsoft Corp., version 16.101.3, Redmond, WA, USA) [25]. Color change (ΔE_00_) was calculated by comparing the color before and after immersion, considering each evaluation period. Moreover, the perceptibility (ΔE_00_ = 1.0) and acceptability thresholds (ΔE_00_ = 3.7) were considered when analyzing color variations [15].

### 2.4. Statistical Analysis

The dependent variable considered was the color difference (ΔE_00_), while GCC thickness and consistency, staining solutions, and immersion time were considered as study factors. The data were analyzed for normality and distribution. Mauchly’s test of sphericity was performed, and Greenhouse-Geisser corrections were applied when sphericity was violated. The 2 consistencies of GCCs were analyzed separately using 3-way repeated measures ANOVAs, followed by the Tukey HSD for multiple comparisons. All analyses were carried out in a statistical software package (SAS OnDemand for Academics SAS Institute Inc., version 9.4, Cary, NC, USA) (α = 0.05).

## 3. Results

**Figure 3:**
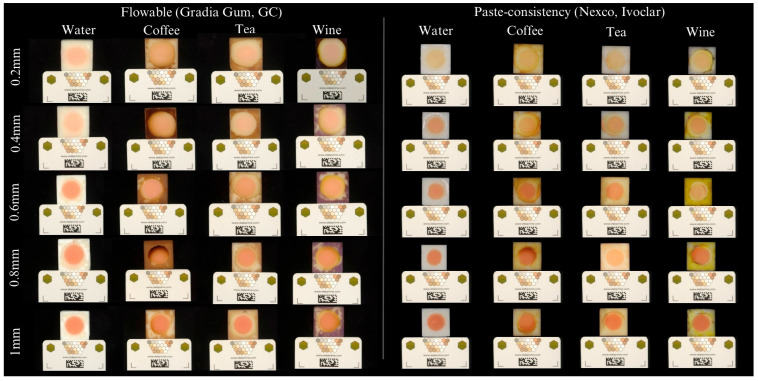
Illustrative example of specimens from the flowable GCC (Gradia Gum; GC) and paste GCC (Nexco; Ivoclar) evaluating each thickness and submersed in each staining solution immersion for 7 days of immersion.

Table 2 and Figure 3 detail the color differences (ΔE_00_) observed in the flowable GCC (Gradia Gum; GC) according to each different thickness and staining solution immersion over different periods. In general, intermediate thicknesses (0.4, 0.6, and 0.8 mm) presented higher color differences (ΔE_00_) than the other remaining thicknesses (0.2 and 1.0 mm), especially in the coffee group (*p* < 0.05) (Table 2). In particular, the highest color differences were observed in the 0.8 mm specimens immersed in coffee solutions for all periods of immersion (*p* < 0.05) (Table 2). In addition, coffee was the most chromogenic agent, inducing significant color changes across nearly all thicknesses and immersion periods (*p* < 0.05) (Table 2). Black tea had a more localized impact, with notable effects in only the 0.4 mm thickness at 2.5 days and in 0.6 mm at 7 days (*p* < 0.05), while water caused no significant alterations (*p* < 0.05) (Table 2). Finally, the highest color differences (ΔE_00_) were observed after 7 days of immersion (*p* < 0.05). However, some specific differences were also observed in the 0.4 and 1 mm thickness specimens after 2.5 days of immersion in tea (Table 2). Nevertheless, most of the color changes observed were considered as clinically perceptible (ΔE_00_ ≥ 0.8) and unacceptable (ΔE_00_ ≥ 1.8) [15].

Table 3 and Figure 3 detail the color differences (ΔE_00_) observed in the paste GCC (Nexco; Ivoclar AG) according to each different thickness and, after immersion, in different staining solutions for different intervals. The greatest color differences (ΔE_00_) were also observed in the 0.8 mm specimens, but, in this case, black tea caused most of the color differences regardless of the period of immersion (*p* < 0.05) (Table 3). Significant color differences were also noted in the 0.4 mm specimens after tea immersion for 5 days and wine immersion for 7 days (*p* < 0.05) (Table 3). Similarly, the 1.0 mm specimens had significant color difference after wine immersion for 2.5 days (*p* < 0.05) (Table 3). Coffee showed a significant chromatic effect only in specific cases, such as the 0.6-mm specimens after 2.5 days of immersion, while the wine solution presented an isolated effect in the 1-mm specimens after 2.5 days (*p* < 0.05) (Table 3). No significant color changes were observed in the control groups of distilled water (*p* < 0.05) (Table 3). Most groups showed no significant differences across the periods of immersion (*p* < 0.05), except the 0.2 mm thick specimens, that presented significant color changes after 7 days of tea immersion, while the 0.4 mm thick specimens showed greater color alteration after 7 days of immersion in both tea and coffee (*p* < 0.05) (Table 3). Nevertheless, most of the color changes observed were considered as clinically perceptible (ΔE_00_ ≥ 0.8) and unacceptable (ΔE_00_ ≥ 1.8) [15].

## 4. Discussion

This study provided one of the first comprehensive investigations into the influence of GCC thickness on color stability by utilizing digital photocolorimetric analysis as a precise and standardized tool to assess color differences over time [20]. The findings revealed that GCC thickness plays a critical role in influencing color behavior, particularly in the flowable formulations. Notably, staining patterns varied according to the 2 different consistencies of GCC. While coffee immersion resulted in the most significant discoloration for flowable GCC, black tea caused greater color alteration for paste GCC. Furthermore, a time-dependent effect on color change was observed in flowable GCC, whereas paste GCC groups showed minimal sensitivity to prolonged exposure (Table 2 and Table 3). These findings demonstrate that both thickness and material consistency critically impact the long-term esthetic performance of GCC-based prostheses. Therefore, clinical decision-making should carefully consider these factors to ensure durable and visually stable outcomes.

In general, greater color alterations (ΔE_00_) were observed in the thicker GCC specimens, particularly in the 0.8 mm specimens of both flowable- and paste GCCs, leading to the rejection of the null hypothesis. This phenomenon may be attributed to the reduced depth of polymerization observed in thicker layers, which results in lower monomer conversion and, consequently, a higher concentration of residual monomers [13]. The presence of unreacted monomers increases the material’s susceptibility to water sorption and pigment uptake, thereby accelerating discoloration over time [13]. From a chemical standpoint, the hydrophilic nature of the residual matrix facilitates the diffusion of staining molecules, while incomplete polymer cross-linking compromises the structural integrity of the network, further promoting pigment penetration [23]. Similar trends have been reported with bulk-fill [13] and conventional composite resins [14], where increased material thickness was associated with greater color instability, reinforcing the role of limited polymerization depth and elevated monomer release in esthetic degradation [13,14]. In addition, non-linear behavior was observed in discoloration, with higher color differences observed at 0.8 mm rather than 1 mm. This unexpected result may be attributed to the complex optical properties of gingival shades and pigment distribution within the GCC [11]. Unlike tooth-colored composites, GCC materials contain specific pink and red pigments designed to mimic soft tissue hues, which interact differently with light [10,11]. As thickness increases, light absorption, scattering, and translucency do not change proportionally, leading to irregular changes in perceived color [11]. Consequently, color perception in these materials does not always increase linearly with thickness, resulting in the observed non-linear discoloration patterns [10]. These results emphasize that composite resin thickness represents a critical determinant for the long-term color stability of GCC-based prostheses. Clinically, this suggests the need for careful control of layer thickness and adequate light-curing protocols to minimize residual monomers and improve the esthetic longevity of the prosthesis [13,23]. However, after 1 month of water storage, no statistically significant differences were detected among thickness groups, which may indicate a post light-curing maturation effect and potential stabilization of the polymeric network over time [14].

The current evidence on color stability of GCC after exposure to staining agents remains scarce, especially when contrasted with the extensive literature on conventional tooth-colored restorative materials [13,14,18,23,24]. Building upon these findings, the present study determined that both the type of staining solution and the physical consistency of GCC play important roles in determining its chromatic behavior. Among the immersion solutions tested, coffee emerged as the most potent chromogenic agent for flowable GCC, inducing significant color alterations across nearly all evaluated thicknesses and time intervals (Table 2). This pronounced effect was consistent with prior reports on conventional composite resins, which have attributed coffee-induced discoloration to its high polyphenol content and strong affinity for polymeric networks [11,15,18]. In contrast, paste GCC showed a higher susceptibility to black tea, whereas coffee and red wine exhibited comparatively selective and less intense effects. Such differences can be explained by the material’s intrinsic characteristics. For instance, the flowable GCC presents greater resin matrix proportion and lower filler loading, increasing water sorption and pigment uptake and making it particularly vulnerable to hydrophilic compounds like those present in coffee [12,22,23]. In contrast, the denser microstructure of paste GCC, featuring a higher inorganic filler content and reduced water sorption, appears to be more prone to interaction with smaller acidic molecules such as those in black tea, which can penetrate and adhere even to minimally porous surfaces [8,12]. Collectively, these observations highlight the combined influence of staining agents and composite resin formulation on the esthetic longevity of GCC-based prosthetic materials.

Notably, this outcome contrasts with those of previous studies [19,24] that identified red wine as the primary staining agent in tooth-colored composite resins—a pattern not replicated in the present investigation. This discrepancy may be attributed to the intrinsic base color of GCCs. As previously suggested [15] and subsequently confirmed [11], the reddish hue of GCCs may partially mask additional discoloration caused by red wine, whose staining effect is typically more pronounced in lighter-shaded resin matrices.

The influence of immersion time on the color stability of GCC revealed distinct material-dependent behaviors. The flowable GCC exhibited a progressive increase in color difference (ΔE_00_) over time, with the greatest discoloration observed at 7 days. This pattern was consistent with that of a previous report [11,22], which attributed higher staining susceptibility in flowable resins to increased water sorption and lower filler content, facilitating chromogen uptake [11]. In contrast, the paste GCC demonstrated better chromatic stability over time. Nevertheless, some deviations were noted as certain specimens exhibited unexpectedly high color change after only 2.5 days of immersion. This may reflect rapid early surface saturation, particularly under the influence of low-molecular-weight and acidic components present in chromatic solutions such as tea. Similar observations have been documented [18], indicating that discoloration in materials with higher resin matrix content can occur rapidly, even before prolonged exposure. In paste CGG, the absence of chromatic changes observed in most thickness groups suggests a higher color stability and resistance to pigment accumulation. These findings were also reported in a previous study [15], concluding that composite resins with higher filler loading and reduced resin content were less susceptible to time-dependent discoloration. The improved performance of paste GCC may also be related to a more stable polymer network, reduced water sorption, and limited interaction with hydrophilic staining agents [23]. In addition, most of the color changes observed were considered as clinically perceptible (ΔE_00_ ≥ 0.8) and unacceptable (ΔE00 ≥ 1.8). These results highlight the importance of material selection for GCC-based prostheses in esthetically demanding patients. Therefore, paste GCC may offer greater long-term color stability, reducing the need for frequent maintenance or replacement in patients with high exposure to staining agents [10]. Conversely, flowable composite resins may require stricter patient counseling regarding dietary habits and professional polishing protocols to maintain optimal esthetics [10].

Limitations of this study included the in vitro design, which could not fully replicate the complexity of the oral environment where factors such as thermal cycling, masticatory forces, enzymatic activity, exposure to different staining agents (smoking, cola, acid beverages, …) and biofilm accumulation can influence material performance—particularly regarding color stability and surface integrity [2,3,10]. Moreover, only 1 type of 3D-printed base resin and 2 GCCs were tested; therefore, the generalizability of these findings to other material classes or formulations remains limited [6,7]. The direct use of wine, in contrast to the standardized preparation of coffee and tea solutions, introduced a methodological inconsistency in the staining protocol. In addition, no artificial aging process was applied, underestimating long-term discoloration and limiting the differentiation between materials. Finally, immersion periods were restricted to 7 days, which may not accurately reflect long-term staining dynamics of several years’ consumption of staining products of the diet. Future studies should incorporate clinically relevant protocols—such as thermocycling, simulated brushing, varied staining agents and methods (e.g., exposure to smoke or other liquid chromogens), and in situ experiments—to more accurately predict clinical performance [12,13].

## 5. Conclusions

This in vitro study demonstrated that the GCC thickness significantly influenced color stability, and increased thickness might be associated with greater color changes over time. Moreover, the flowable GCC exhibited higher susceptibility to staining, particularly when immersed in coffee solutions, and demonstrated progressive discoloration with time. In contrast, paste GCC maintained greater chromatic stability, likely because of its higher filler content and reduced water sorption.

## Figures and Tables

**Table 1:** Composition of materials used.

Materials	Manufacturer	Batch Nº	Composition *
P Pro Resin	Institut Straumann AG	231,568	Acrylic resin, urethane dimethacrylate (UDMA), diacrylate, phosphine oxide, diphenyl (2,4,6-trimethylbenzoyl) (TPO)
Gradia Plus Gum	GC Europe (Louvain, Belgium)	2,201,261	Urethane dimethacrylate (UDMA) (25−50%), dimethacrylate component (5−10%), dimethacrylate (1−5%), trimethacrylate (1−5%) UV-light absorber (1−5%)
SR Nexco Paste	Ivoclar AG (Schaan, Liechtenstein)	Z047PF	DMA (17–19 wt%); copolymer and silicon dioxide (82–83 wt%), stabilizers, catalysts, pigments, 10–100-nm inorganic fillers (64–65 wt%)

* Information obtained from safety data sheets provided by manufacturers (data sheets provide potential component ranges for each product but do not guarantee specific composition).

**Table 2:** Mean ± standard deviation of color differences (ΔE_00_) observed in flowable GCC (Gradia Gum; GC) according to each different thickness and after immersion in different staining solutions over different periods.

	2.5 Days				5 Days				7 Days			
Thickness (mm)	Water	Wine	Coffee	Tea	Water	Wine	Coffee	Tea	Water	Wine	Coffee	Tea
0.2	1.65 ± 1.03 Aa	2.54 ± 1.09 ABab*	3.72 ± 2.27 Ab*	2.38 ± 1.66 Aab	2.00 ± 1.38 Aa	3.66 ± 2.16 Aab**	5.61 ± 3.37 Ab**	2.70 ± 1.17 Aa	2.23 ± 0.97 Aa	4.36 ± 2.43 Aa**	6.78 ± 4.042 Ab***	2.94 ± 0.82 ABa
0.4	1.17 ± 0.86 Aa*	2.03 ± 1.03 Aab	4.09 ± 0.30 Abc*	6.09 ± 0.98 Bc*	1.77 ± 0.93 Aa*	2.81 ± 1.33 Aab	6.15 ± 1.17 Ac**	4.02 ± 0.96 Abc**	2.93 ± 1.35 Aa**	2.80 ± 1.59 Aa	7.32 ± 0.816 Ab***	3.09 ± 1.03 ABa***
0.6	2.62 ± 1.02 Aa	4.42 ± 3.57 ABa	5.25 ± 2.12 ABa*	4.31 ± 4.55 ABa*	3.13 ± 0.95 Aa	4.44 ± 3.40 Aa	5.63 ± 2.55 Aa*,**	3.23 ± 2.43 Aa*,**	2.90 ± 0.72 Aa	3.86 ± 3.99 Aab	6.23 ± 3.048 Ab**	5.23 ± 5.27 Bab**
0.8	1.94 ± 0.43 Aa	4.79 ± 4.19 Bb*,**	7.91 ± 1.98 Bc*	2.63 ± 0.56 Aab	2.50 ± 0.63 Aa	4.96 ± 4.10 Aa*	7.95 ± 2.87 Ab*	2.49 ± 0.73 Aa	2.49 ± 0.77 Aa	3.99 ± 4.80 Aa**	10.37 ± 2.849 Bb**	2.13 ± 0.67 Aa
1.0	1.79 ± 1.13 Aa*	2.70 ± 2.84 ABab*	5.62 ± 3.47 ABc*	4.63 ± 2.72 ABbc*	2.93 ± 1.00 Aa**	3.36 ± 2.25Aab*,**	5.48 ± 1.92Ab*	4.40 ± 2.40 Aab*	2.39 ± 1.24 Aa*,**	3.76 ± 3.33 Aa**	6.62 ± 2.73 Ab**	2.47 ± 1.39 ABa**

Different uppercase letters indicate significant differences between different GCC thickness in each staining solution of each period of immersion (*p* < 0.05). Different lowercase letters indicate significant differences between different staining solutions in each GCC thickness of each period of immersion (*p* < 0.05). *, **, *** indicate significant differences between different periods of immersion in each GCC thickness of each staining solution (*p* < 0.05).

**Table 3:** Mean ± standard deviation of the color differences (ΔE_00_) observed in paste GCC (Nexco; Ivoclar AG) according to each different thickness and after immersion in different staining solutions over different periods.

	2.5 Days				5 Days				7 Days			
Thickness (mm)	Water	Wine	Coffee	Tea	Water	Wine	Coffee	Tea	Water	Wine	Coffee	Tea
0.2	2.14 ± 0.77 Aa	2.67 ± 0.83 Aa	3.79 ± 1.79 ABa	3.69 ± 0.68 Aa*	2.42 ± 1.78 Aa	2.81 ± 0.91 Aa	3.34 ± 2.44 Aab	5.03 ± 1.00 ABCb*,**	2.48 ± 1.13 ABa	2.85 ± 0.98 Aa	4.33 ± 2.51 Aab	5.48 ± 0.52 ABb**
0.4	2.30 ± 0.90 Aa	2.79 ± 0.64 Aa*	3.01 ± 1.49 Aab	4.84 ± 2.01 Ab*	3.08 ± 1.18 Aa	3.98 ± 1.22 Aa*,**	4.11 ± 1.59 Aa	6.69 ± 2.66 Ab**	3.45 ± 2.55 Ba	4.94 ± 2.50 Bab**	3.62 ± 2.20 Aa	6.24 ± 2.67 Bb**
0.6	1.93 ± 0.54 Aa	3.65 ± 2.32 Ab	3.87 ± 2.06 ABab	3.57 ± 1.50 Aab	1.61 ± 0.68 Aa	3.43 ± 1.75 Aab	3.84 ± 1.94 Ab	4.56 ± 0.72 BCb	1.29 ± 0.44 Aa	4.47 ± 2.38 ABb	5.15 ± 2.62 Ab	4.78 ± 1.10 ABb
0.8	1.88 ± 1.174 Aa	5.59 ± 2.69 Bb*	5.34 ± 2.03 Bb*	10.28 ± 2.04 Bc*	2.18 ± 1.07 Aa	3.19 ± 1.28 Aa**	4.04 ± 2.21 Aa**	6.14 ± 1.21 ABb**	2.20 ± 1.44 ABa	4.15 ± 2.06 Aba*,**	3.28 ± 1.92 Aa**	6.80 ± 2.55 Bb**
1.0	2.64 ± 1.4 Aa	5.46 ± 2.67 Bb*	4.06 ± 1.90 ABab	4.11 ± 1.15 Aab	2.00 ± 1.08 Aa	3.43 ± 1.42 Aa**	2.98 ± 2.17 Aa	3.47 ± 0.70 Ca	1.74 ± 0.75 ABa	3.00 ± 1.69 ABab**	3.22 ± 1.78 Aab	4.15 ± 0.74 Ab

Different uppercase letters indicate significant differences between different GCC thickness in each staining solution of each period of immersion (*p* < 0.05). Different lowercase letters indicate significant differences between different staining solutions in each GCC thickness of each period of immersion (*p* < 0.05). *, ** indicate significant differences between different periods of immersion in each GCC thickness of each staining solution (*p* < 0.05).

## Data Availability

The original contributions presented in this study are included in the article. Further inquiries can be directed to the corresponding author.
